# COVID-19 outbreak, herd immunity formation, and future public health strategies

**DOI:** 10.4178/epih.e2021071

**Published:** 2021-09-16

**Authors:** Youngtaek Kim, Yoon Hyung Park

**Affiliations:** 1 Public Health Medical Service Office, Chungnam National University Hospital, Daejeon, Korea; 2 Department of Preventive Medicine, Soonchunhyang University College of Medicine, Cheonan, Korea

**Keywords:** Stringency index, Mitigation measure, COVID-19 herd immunity

## Abstract

In Korea, where the successful control of the coronavirus disease 2019 (COVID-19) epidemic have been implemented by the follow-up survey management (containment) of COVID-19-infected persons, the number of infected persons has increased rapidly, and a re-epidemic trend is emerging. The Korean government is strengthening epidemic prevention activities, such as increasing the social distance in the metropolitan area to four levels and increasing the vaccination rate. The public has been complaining of dissatisfaction with the atrophy of socioeconomic activities and of distrust of epidemic prevention policies. Australia started with an incidence similar to that of Korea, but its social activities are more flexible than those of Korea, where the incidence is maintained at approximately 0.1 per 100,000 people. In comparing the differences between both countries in terms of the Oxford COVID-19 Government Response Tracker Stringency Index, it was found that Australia effectively regulates the number of infected cases by high-intensity intermittent mitigation and the subsequent allowance of social activities. Korea has also recommended a high-intensity intermittent mitigation policy as in Australia until community herd immunity via vaccination is formed.

## A REVIEW OF THE COVID-19 RESURGENCE AND ITS FACTORS

Korea has successfully implemented public health epidemic control strategies, such as wearing a mask and social distancing, to maintain the daily incidence of confirmed coronavirus disease 2019 (COVID-19) cases at a level of 0.5 to 2.0 per 100,000 population. However, the introduction of the delta (δ) variant and delays in vaccination made Korea enter the fourth wave of the COVID-19 epidemic in early July 2021. This has brought about concerns regarding about the community pandemic crisis experienced by the United States and European countries. Accordingly, from July 12, 2021, the highest level of prevention and control measures, ‘the fourth stage of social distancing,’ was implemented in the metropolitan area. Israel is a representative country that focuses on achieving herd immunity through vaccination rather than through a public health epidemic control strategy, and as of the end of March 2021, 60% of the total population has completed primary vaccinations. Thanks to the success of these vaccinations, the daily incidence of COVID-19 cases decreased to less than 100 people; however, from the end of June, it again exceeded 100 people per day. This shows that it is difficult to find a permanent resolution for the COVID-19 epidemic with a vaccination rate of 60%, and re-spreading is possible if public health epidemic control strategies, such as wearing a mask and social distancing, are not strictly implemented.

After the second wave of the pandemic was controlled in Germany, the easing of social prevention and control triggered the third wave pandemic. Schuppert et al. [1] found that during the second wave in Germany at the end of 2020, partial lockdown (mitigation) did not control the pandemic, whereas the lockdown of the entire society did.

There are several factors involved in the COVID-19 resurgence. First, in terms of the pathogenic factor, the transmission of recently discovered variants has increased. According to recent United Kingdom variant monitoring results, compared to the variant first reported in Wuhan, China, the transmission of the alpha (α) variant increased by 50%, compared to which that of the δ variant increased by 60% [2,3]. It is known that the longer the epidemic period and the greater the number of infected persons, the higher the possibility of the appearance of variants of severe acute respiratory syndrome coronavirus 2 (SARS-CoV-2) [4].

Second, in terms of the vaccination factor, the herd immunity percentage required to contain the community outbreak of COVID-19 to the level of eradication was predicted to be approximately 60% at the beginning of the pandemic (1-1/basic reproduction number [Ro], %, Wuhan Ro 2.6, 61.5%). When estimating the preventive effect of the vaccine at 60–80%, we found that 80–90% of the entire population needs to be vaccinated to reach herd immunity. If the variant becomes a major infection source worldwide, it is impossible to form herd immunity even if 90% of the population is vaccinated with the currently developed vaccines. In a situation where the duration of the effectiveness of the vaccine is uncertain, the longer the period to reach the target of the overall vaccination rate for the entire population, the shorter the period of maintaining the established herd immunity, especially compared to the duration of the individual’s vaccine preventive effect. As in the human corona virus reinfection cases reported by Edridge et al. [5] and in COVID-19 reinfection cases, if the vaccine effect lasts only 6 months or 12 months. and the vaccination period is longer than 8 months, herd immunity is not achieved, or the maintenance period can be significantly shortened even if herd immunity is formed (herd immunity maintenance period=duration of vaccine immunity - period of simultaneous vaccination).

Third, as can be seen from Israel’s experience, easing prevention and control measures, such as wearing a mask and social distancing, without forming herd immunity will result in a resurgence in the community.

## COMPARISON OF COVID-19 PREVENTION AND CONTROL POLICIES IN AUSTRALIA AND KOREA THAT SUPPRESSED THE COVID-19 EPIDEMIC

Kissler et al. [6] suggested that a high-intensity intermittent lockdown (intermittent mitigation) in an insufficiently vaccinated population could suppress the epidemic. Australia implemented a high-intensity intermittent lockdown (intermittent mitigation) as proposed by Kissler et al. [6] while vaccination was insufficient. Australia has suppressed less than 100 people per week and less than 0.1 per 100,000 daily until just before winter 2021, after the resurgence in mid-2021. This can be a successful case of a sustainable response to COVID-19, as proposed by Mulheirn et al. [7].

Hale et al. [8] developed a standard index to measure the infection prevention and control intensity of countries responding to COVID-19 from the beginning of 2020 to the present, measuring the results for each country on a scale of 1 to 100 and releasing them daily. The World Health Organization (WHO) provides this index through its official website. The Oxford COVID-19 Government Response Tracker (OxCGRT) index consists of 13 items in three domains: social distancing, such as school closures, restrictions on gatherings, and mobility; patient treatment; isolation tracking management; medical systems in place; and economic support. These reflect almost all COVID-19 response policies that countries can mobilize [7].

**Figure 1.**
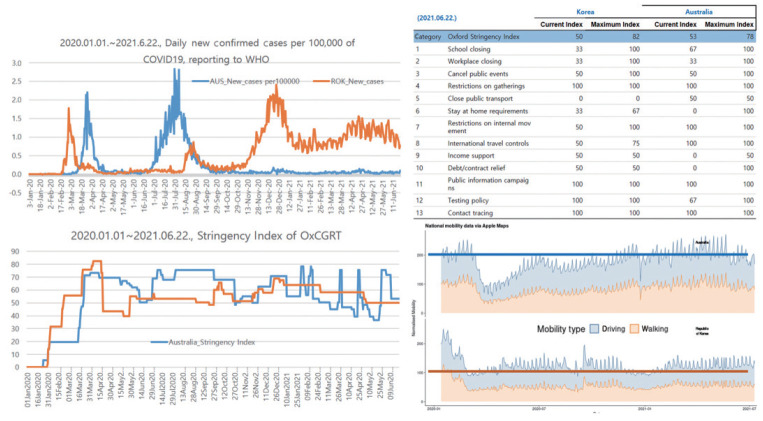
Trends of daily new confirmed cases per 100,000 of COVID-19, Stringency Index of OxCGRT, and mobitiy via Apple Maps in Australia (AUS) and Republic of Korea (ROK). WHO, World Health Organization; OxCGRT, Oxford Coronavirus Disease Government Response Tracker. Source from: World Health Organization. WHO coronavirus (COVID-19) dashboard [9]; Hale T, et al. Oxford COVID-19 government response tracker [10].

In observing the daily incidence trend, intensity of prevention and control as measured via the OxCGRT, and mobile social interaction in Australia, and in comparing these with those of Korea, we found the following characteristics (Figure 1) [9,10].

First, the size and pattern of the epidemic in each country were quite similar, such as suppressing the daily incidence to less than 0.1 per 100,000 population in a sporadic pattern, from the first epidemic wave caused by overseas inflow (March 2020 in Korea, April 2020 in Australia) to the resurgence in winter (December 2020 in Korea, August 2020 in Australia).

Second, Australia has controlled the daily incidence to below 0.1 per 100,000 population since September 2020, when the winter resurgence ended, while Korea has not been able to control the daily incidence to less than 1.0 per 100,000 population since January 2021, after experiencing a winter resurgence. For 173 days from January 1 to June 22, 2021, Australia had a daily average of 0.045 (minimum, 0.004; maximum, 0.145), with 167 days below 0.1 (96.5%, 167/173) and no more than 0.5 (0 days). In Korea, the daily average was 1.027 (minimum, 0.561; maximum, 2.009), with 0 days below 0.1, 173 days above 0.5, and 82 days above 1.0 (47.4%, 82/173). In terms of the daily pattern and scale of incidence, it can be said that Korea had ten times more cases than did Australia.

Third, when comparing the OxCGRT from the University of Oxford website (https://covidtracker.bsg.ox.ac.uk/): We have produced four indices that aggregate the data into a single number from 0 to 100, as published by the WHO for 173 days from January 1 to June 22, 2021, Australia had a daily average of 54.9 (minimum, 36.6; maximum, 78.2), 41 days above 70, and 61 days below 50 (35.3%, 61/173). In Korea, the daily average was 57.9 (minimum, 50.0; maximum, 67.6), with 0 days above 70 and 0 days below 50. Korea gradually declined from a daily average of 66 on January 1, 2021 to that of 50 on June 22, 2021. It is presumed that the social acceptance of the government’s prevention and control measures has decreased (Table 1).

Fourth, the OxCGRT Stringency Index is associated with the occurrence after 2 weeks. The higher the OxCGRT Stringency Index before the first week, the higher the probability that the infected case count will decrease after 2 weeks. Upon investigating Korea’s COVID-19 prevention and control response and outbreak trend from January 1 to June 22, 2021 using the OxCGRT Stringency Index, with daily incidence increased by more than 0.5 per 100,000 population, unless Korea’s response to COVID-19 exceeds the OxCGRT Stringency Index of 58, we concluded that it is unlikely that it will turn into a decrease in the occurrence trend.

Fifth, according to the normalized mobility presented by the WHO for 173 days from January 1, to June 22, 2021, Australia had a vehicle mobility index of approximately 200 and a walking mobility index of approximately 100, and Korea had approximately 100 and 50, respectively. According to these observations, it can be assumed that the amount of social interaction in Australia is twice that in Korea.

Finally, despite the sizes and transmission patterns of outbreaks in Korea and Australia being similar in the beginning, due to the difference in COVID-19 prevention and control policies between the two countries from January to June 2021, Australia maintains an incidence of 0.1 lower than that of Korea, even though social activities in Australia have doubled compared to those of Korea. This is thought to be due to the short-term high-intensity social control (intermittent mitigation) implemented in Australia and the allowance of relatively free activities after that. Korea also needs to strengthen social prevention and control to at least 58 in the OxCGRT Stringenxy Index to control COVID-19 cases, which have resurged since July 21, 2021. In addition, control responses to the OxCGRT Stringency Index of 68 or higher are needed, as the daily number of cases was reduced to less than 0.1 per 100,000 population in the early 2020 epidemic wave. After controlling the resurgence in July, it is necessary to review the policy of short-term high-intensity social control (intermittent mitigation) that is followed by relatively free activities, as in Australia.

## SUGGESTIONS FOR EFFECTIVE COVID-19 PREVENTION AND CONTROL POLICIES

COVID-19 infection has already been identified as a risk factor for severe cardiovascular and cerebrovascular diseases less than a year after the pandemic [11].

The SARS-CoV-2 variants are transmitted much more quickly than expected, and the effectiveness of vaccines is likely to last for a shorter duration than expected. Since transmission increases with the appearance of each variant, it is impossible to form herd immunity at the eradication level with the current vaccines. In addition, repeat vaccination is required due to limitations on the vaccine’s effectiveness; however, this is a dilemma that can cause an increase in adverse reactions.

Given that the burden of disease of COVID-19 is not limited to the acute phase, and the long-term burden of disease of COVID-19 and the long-term effects of adverse reactions after vaccination are uncertain, alternatives should be implemented to minimize the amount of infection, to reduce side effects such as social distancing and the number of vaccinations, and to decrease the burden of direct and indirect and long-term and short-term burdens of diseases caused by COVID-19. In terms of health and socioeconomic perspectives, intermittent mitigation, which implements the highest intensity measures within 2 weeks and allows the public to engage in relatively free activities for approximately 2 months after that, is considered the best way for social quarantine.

**Figure 2.**
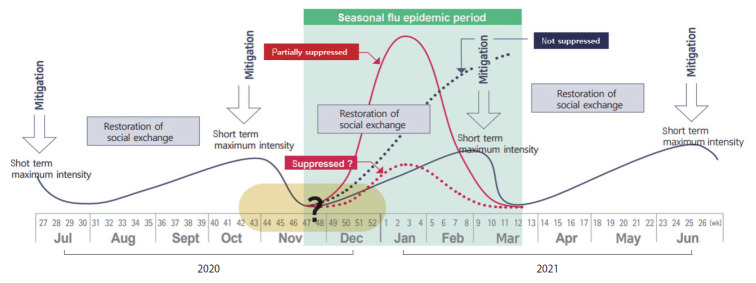
Action plan of intermittent mitigation. Short-term (7 days, >serial interval) maximum intensity (everybody, stay home) mitigation (1st) to minimize residual infection; Short-term high-intensity mitigation immediately before entering the seasonal flu epidemic period (mid-November to mid-March), repeat; Restoration of social exchange (releasing social distancing), suppression of the increase in the amount of residual infection (early monitoring, forced wearing of masks, etc.).

Figure 2 is a schematic diagram of the authors’ argument for implementing the highest intensity of intermittent mitigation required to counter the recent re-increase in COVID-19 incidence and to prepare for the anticipated resurgence in the upcoming winter.

The solid red line is the content that predicts the prevalence when partial mitigation is implemented, and the dotted blue line is the content that predicts the prevalence when mitigation is not implemented. The dotted red line predicts when mitigation is well-managed. The solid blue line shows the case of Australia, where the epidemic is controlled by implementing intermittent mitigation.

Referring to the case of Australia, a four-step method is proposed that can be reviewed as a COVID-19 prevention and control measure in preparation for winter in Korea.

(1) First of all, the highest intensity of mitigation measures (everybody stays at home) are fully implemented for the minimum period for the intervention effect to appear (> serial interval of COVID-19, 4-6 days or more), thereby reducing the amount of residual infection as much as possible.

(2) The next step is to discontinue the policy of social distancing, which has severe side effects, to restore social interaction to a level close to that before the COVID-19 outbreak, and to continue as such until the COVID-19 incidence reaches a certain level (e.g., 1 per 100,000 population per day).

(3) In order to ensure that the restoration period of social interaction is at least 2 months to 3 months, the increase rate in the incidence of COVID-19 should be suppressed as much as possible by strengthening containment through early detection and mobilizing preventive management measures, such as wearing a mask at all times.

(4) If the occurrence of COVID-19 exceeds a certain threshold, the highest intensity mitigation measures are retaken, and social interaction is restored.

## LIMITATIONS

Even though Australia and Korea have similar vaccination rates, they differ in seasons, total population, population density, and social behavior. Therefore, comparing the two countries superficially and benchmarking to suggest alternatives will produce limitations in accuracy. Despite these limitations, the introduction of Australia’s successful restrictions was presented as a reference for Korea’s COVID-19 prevention and control policies.

## CONCLUSION

It is ideal to secure sufficient vaccines and implement them as quickly as possible instead of introducing long-term vaccinations. Since Korea has an infrastructure that allows more than 50 million vaccinations a month, this is a feasible policy in Korea. This will maximize the duration and effect of herd immunity and reduce the number of repeated vaccinations as much as possible.

In addition, the highest intensity of intermittent mitigation will minimize the amount of infection, thereby extending the period of restoration of social exchanges and preparing for the winter twindemic.

### Ethics statement

This study was written by reviewing published research papers, it is exemption to deliberation by the institutional review board.

## Figures and Tables

**Table 1.** Difference of prevalence (daily new confirmed cases per 100,000 of population) and response (OxCGRT Stringency Index) of COVID 19 between Australia and Korea

Period in 2021 (Jan 1-Jun 22, 173 d)		Australia	Korea
Daily new confirmed cases per 100,000 of population reporting to WHO (P)	Mean of P’s	0.045	1.027
	Maximum of P’s	0.145	2.009
	Minimum of P’s	0.004	0.561
	No. of days if p>1.0	0	82, 4.7% (82/173)
	No. of days if p>0.5	0	173
	No. of days if p<0.1	168, 1.0% (167/173)	0
OxCGRT Stringency Index, daily score (S)	Mean of daily OxCGRT’s	54.9	57.9
	Range (maximum of daily OxCGRT’s – minimum of daily OxCGRT’s)	41.6 (78.2–36.6)	17.6 (67.6–50.0)
	No. of days if S >70	41 (23.7%, 41/173)	0
	No. of days if S <50	61 (35.3%, 61/173)	0
Normalized mobility via Apple Maps	Walking	Approximately 100	Approximately 50
	Driving	Approximately 200	Approximately 100

Source from: World Health Organization. WHO coronavirus (COVID-19) dashboard [9]; Hale T, et al. Oxford COVID-19 government response tracker [10]. WHO, World Health Organization; OxCGRT, Oxford Coronavirus Disease Government Response Tracker.

## SUPPLEMENTARY MATERIALS

Korean version is available at https://www.e-epih.org/.
