# Supplementary material for: COVID-19 outbreak, herd immunity formation, and future public health strategies
Source: Epidemiol Health. 2021 Sep 16;43:e2021071. doi: 10.4178/epih.e2021071 (PMC8666682; doi:10.4178/epih.e2021071)
Supplement: Supplementary file 1 [file epih-43-e2021071-suppl.docx]

**Perspective**

**코로나19 발생 및 집단면역 형성 전망과 향후 공중보건 전략**

**COVID-19 outbreak, herd immunity formation and future public health strategies**

**Young Taek Kim^1^, Yoon Hyung Park^2^**

**^1^Chungnam National University Hospital; ^2^College of Medicine, Soonchunhyang University**

**Correspondence: Yoon Hyung Park, MD, PhD.**

**Department of Preventive Medicine, Soonchunhyang University, 31 Soonchunhyang 6-gil, Cheonan Si, Chungnam, 31151, Republic of Korea. Email:** [**parky@sch.ac.kr**](mailto:parky@sch.ac.kr)

요약 Abstract

COVID-19 감염자에 대한 추적조사관리(containment)를 중심으로 성공적인 방역을 시행하던 한국에서 최근 감염자가 급증 하는 등 재 유행 확산추세가 나타나고 있다. 한국정부에서는 수도권 지역에 사회적 거리 두기를 4단계로 상향 조정하고 백신 접종에 속도를 내는 등 방역활동을 강화하고 있다. 국민들은 사회경제활동 위축에 따른 불만과 방역정책에 대한 불신과 따른 피로감을 호소하고 있다. 호주는 한국과 유사한 발생률로 시작하였으나 사회적 활동이 한국보다 유연하면서 발생률을 10만명 당 0.1 정도로 유지하고 있다. 두 나라의 차이를 OxCGRT Stringency Index of Government Response를 중심으로 비교한 결과 호주는 고강도의 intermittent mitigation 과 그 이후 사회적 활동 허용 등으로 효과적으로 감염양을 조정하고 있는 것으로 조사되었다. 한국도 현 단계에서 백신접종으로 지역사회 집단면역이 형성되기 전까지 호주와 같이 고강도의 intermittent mitigation정책을 검토할 것을 권고하였다.

Key word : Stringency Index, mitigation measure, COVID-19 herd immunity

**1. COVID-19 재 유행과 요인 고찰**

한국은 그 동안 마스크 착용, 사회적 거리두기 등과 같은 공중보건학적 유행통제 전략을 성공적으로 추진하여 COVID-19 확진자 일일 발생률을 인구10만 명당 0.5~2.0 수준으로 유지할 수 있었다. 그러나 델타 변이 종 등의 유입 및 예방접종 지연 등으로 인하여 2021년 7월 초 4차 유행 단계로 접어들었으며, 미국과 유럽 국가들이 경험한 지역사회 대유행 위기를 우려해야 할 상황이다. 이에 2021년 7월 12일부터는 수도권에 최고 수준인 ‘사회적 거리두기 4단계’ 방역조치가 전격 실시되었다. 이스라엘은 공중보건학적 유행통제 전략보다 예방접종을 통한 군집면역 달성에 역점을 둔 대표적인 국가로서 2021년 3월말 기준 전 국민의 60%가 ~~에~~ 기초접종을 완료한 바 있다. 이러한 예방접종의 성과에 힘입에 코로나19 일일 발생자는 100명 미만 수준으로 감소하였으나, 6월 하순 부터는 다시 일일 100명을 넘어섰다. 이러한 이스라엘 사례는 60% 수준의 예방접종률로는 코로나19 유행 종식을 달성하기는 어려우며, 마스크 착용 및 사회적 거리두기와 같은 공중보건학적 유행통제 전략이 느슨해질 경우 재확산이 가능함을 보여주고 있다.

독일은 2차 대유행이 통제된 이후 사회방역 완화로 인해 제3차 대유행을 촉발하는 현상이 발생하였다. Andreas Schuppertet 등은 2020년 말 독일에서 발생한 제2차 대유행 기간 동안 부분적인 봉쇄(Mitigation)로는 대유행을 통제하지 못하였고, 사회 전체적인 봉쇄로 대유행을 통제하였다고 보고하였다.(1)

COVID-19 재 유행 요인을 살펴보면,

첫째, 병원체 요인을 보면, 최근에 발견되는 변이 종 들의 전파력이 증가하였다. 최근 영국의 변이 종 모니터링에 의하면 중국 우한에서 처음 보고된 종 보다 α 는 전파력이 50% 증가, δ는 α보다 전파력이 60% 증가하였다(2)(3). 유행기간이 길어지고 감염량이 많아질수록 SARS CoV 2의 변이 출현 가능성이 높아진다고 알려져 있다(4).

둘째, 예방접종 요인을 보면, COVID-19 지역사회 유행을 퇴치수준으로 억제하기 위해 필요한 집단면역은, 대유행 초기에는 60% 정도로 예측되었고(1-1/Ro : 기본재상산 지수, %, Wuhan Ro 2.6, 61.5%), 백신의 예방효과가 60~80%로 추정할 때 전 국민의 80~90%가 접종해야 도달 할 수 있다. 변이종이 전 세계에서 주요 감염원이 될 경우, 현재 개발된 백신으로는 전 국민의 90%를 접종하더라도 집단면역 형성은 불가능하다. 백신의 효과 지속기간이 불확실한 상황에서 전 국민에 대한 전체 접종률 목표에 도달하는 기간이 길어질수록 형성된 집단면역의 유지기간은 개인의 백신 예방효과 지속기간 보다 짧아질 수 있다. Edridge (5)등이 보고한 HCV 재감염 사례들과 COVID-19 재감염 사례들에서 보듯이 백신 효과 지속기간이 6개월 또는 12개월 밖에 되지 않고 현재와 같이 접종기간이 8개월 이상 되면 집단면역이 형성되지 않거나 형성되더라도 유지기간이 상당히 단축될 수 있다(집단면역유지 기간 = 백신 면역지속기간 – 일제접종기간).

셋째, 이스라엘의 경험에서 확인할 수 있듯이 집단면역 형성 없이 마스크 상시 착용과 거리 두기 등 방역을 완화할 경우 지역사회 재 유행을 촉발하게 된다.

2. COVID-19 유행을 억제한 호주와 한국 방역정책 비교

Kissler and Lipsitch et al 등은 예방접종이 미흡한 상태에서 고강도의 간헐적 봉쇄(intermittent mitigation)가 유행을 억제할 수 있다고 제시하였다.(6) 호주는 예방접종이 미흡한 상태에서 Kissler and Lipsitch et al 등이 제안한 고강도의 간헐적 봉쇄(intermittent mitigation)를 시행하였다. 호주는 2020년 중반 겨울철 재 유행 이후, 2021년 겨울철 직전까지 주당 100명 이하, 일 발생 인구 10만 명당 0.1 이하로 억제하였다. 이는 TONY BLAIR INSTITUTE FOR GLOBAL CHANGE 가 제기한 지속 가능한 방역의 성공사례라고 할 수 있다(7)

Thomas Hale 등은 2020년 초부터 현재까지 COVID-19에대응하는 국가의 방역강도를 측정하는 표준 지표를 개발하여 국가별 측정결과를 1점~100점으로 측정하여 일단위로 공개하고 있다(OxCGRT Stringency Index of Government Response). WHO는 공식 인터넷 사이트를 통해서 이 지표를 제공하고 있다. OxCGRT 지표는 학교 폐쇄, 모임 및 이동 제한 등 사회적 거리 두기, 환자 치료 및 격리 추적 관리와 의료체계, 경제적 지원 등 3개 영역 13개 항목으로 구성되어 있고, 국가가 동원 가능한 거의 모든 COVID-19 대응정책을 반영하고 있다.(8)

호주의 일 발생 추이와, OxCGRT에 의한 방역강도와 모바일 사회교류를 관찰하고 현재 한국과 비교하여 보면 다음과 같은 특징들이 있다.(9)(10) (그림 1)

첫째. 해외유입에 의한 첫 유행 이후(한국 2020.3, 호주 2020.4.) 겨울철 재 유행까지는(한국 2020.12., 호주 2020.8.) 우리나라와 호주는 일 발생 인구 10만 명당 0.1 이하로 산발적 발생양상으로 억제하는 등 발생 규모와 양상이 상당히 유사하였다.

둘째. 호주는 겨울철 재 유행이 끝난 2020년 9월부터 일 발생을 인구 10만 명당 0.1 이하로 통제하고 있는 반면, 한국은 겨울철 재 유행을 겪고 난 2021년 1월부터 일 발생을 인구 10만 명당 1 이하로 통제하지 못하고 있다. 2021년 1월 1일부터 2021년 6월 22일까지 173일 동안, 호주는 일 평균 0.045(최소 0.004, 최대 0.145)이었고 0.1이하 일수 167일(97%, 167/173), 0.5 이상일수는 없었다(0일). 한국은 일 평균 1.027(최소 0.561, 최대 2.009)이었고 0.1이하 일수 0일, 0.5 이상일수 173일, 1.0이상 일수 82일(47%, 82/173))이었다. 일 발생 양상과 규모로 볼 때, 한국은 호주보다 10배 더 많이 발생하고 있다고 할 수 있다.

셋째. 2021년 1월 1일부터 2021년 6월 22일까지 173일 동안 WHO가 게시한 OxCGRT Stringency Index(1~100)를 비교해 보면, 호주는 일 평균 54.9(최소 36.6, 최대 78.2) 이었고 70 초과일수 41일, 50 미만 일수 61일(35%, 61/173))이었다. 한국은 일 평균 57.9(최소 50.0, 최대 67.6)이었고 70 초과일수 0일, 50 미만 일수 0일이었다. 한국은 2021년 1월1일 일 평균 66에서 2021년 6월22일 일 평균 50으로 단계적으로 감소하는 양상을 보였다. 원인으로 정부의 방역 조치에 대한 사회 수용도가 낮아진 것으로 추정된다.(Table 1)

Table 1. Difference of prevalence(Daily New Confirmed Cases per 100,000 of Population) and response(OxCGRT Stringency Index) of COVID 19 between Australia and Republic of Korea

| Period 2021.01.01.~6.22.(173 days) | | Australia | Republic of Korea |
| --- | --- | --- | --- |
| Daily New Confirmed Cases  per 100,000 of Population  reporting to WHO(P) | Mean of P’s | 0.045 | 1.027 |
|  | Maximum of P’s | 0.145 | 2.009 |
|  | Minimum of P’s | 0.004 | 0.561 |
|  | Number of Days if P>1.0 | 0 | 82, 47%(82/173) |
|  | Number of Days if P>0.5 | 0 | 173 |
|  | Number of Days if P<0.1 | 167, 97%(167/173) | 0 |
| OxCGRT Stringency Index,  Daily Score(S) | Mean of Daily OxCGRT’s | 54.9 | 57.9 |
|  | Range(Maximum of Daily OxCGRT’s – Minimum of Daily OxCGRT’s | 41.6(78.2 – 36.6) | 17.6(67.6 – 50.0) |
|  | Number of Days if S >70 | 41(24%, 41/173) | 0 |
|  | Number of Days if S <50 | 61(35%, 61/173) | 0 |
| Normalized Mobility  via Apple Maps | Walking | around 100 | around 50 |
|  | Driving | around 200 | around 100 |
| Source | Source: WHO COVID-19 Explorer. https://worldhealthorg.shinyapps.io/covid/ , Oxford Stringent Index Government Response, <https://covidtracker.bsg.ox.ac.uk/> | | |

넷째, OxCGRT Stringency Index는 2주 후의 발생과 연관되어 있다. 1주 전의 OxCGRT Stringency Index가 증가할수록 2주후에 감염발생이 감소할 가능성이 높아진다. 인구 10만 명당 일 발생이 0.5 이상 증가한 한국의 2021년 1월 1일부터 2021년 6월 22일까지 방역 대응과 발생추이를 OxCGRT Stringency Index를 이용하여 살펴보면 한국의 방역 대응이 OxCGRT Stringency Index 58보다 초과하는 수준이 아니면 발생 추이감소로 전환될 가능성이 낮다.

다섯째, 2021년 1월 1일부터 2021년 6월 22일까지 173일 동안 WHO에 게시된 Normalized Mobility를 보면, 호주는 차량 이동 지수 200 내외, 도보 이동 지수 100내외였고, 한국은 각각 100과 50 내외였다, 이러한 관측결과에 따르면 호주의 사회 교류 량은 한국보다 2배 많다고 볼 수 있다.

결론적으로, 초기에는 한국과 호주의 발생규모와 전파양상이 유사하였으나 2021년 1월부터 6월까지 양국의 방역정책의 차이에 따라 호주는 국민의 사회활동이 한국보다 2배 증가했는데도 불구하고 발생률은 한국보다 낮은 0.1 수준을 유지하고 있다. 이는 호주에서 시행하는 단기적 고강도 사회통제(intermittent mitigation)와 그 후 비교적 자유로운 활동 허용 정책 때문이라고 추정된다. 한국도 21년 7월 재 유행하고 있는 코로나 19의 통제를 위해서 사회방역을 OxCGRT Stringenxy Index 58 이상으로 강화할 필요가 있다. 추가하여 2020년 초기 유행 시 일 발생 인구 10만 명당 0.1 이하로 감소시켰던 것과 같이 OxCGRT Stringenxy Index 68 이상의 방역대응이 필요하다. 7월 재 유행을 통제하고 난 이후에는 호주와 같이 단기적 고강도 사회통제(intermittent mitigation)와 그 후 비교적 자유로운 활동 허용 정책을 검토해야 한다고 생각한다.

**
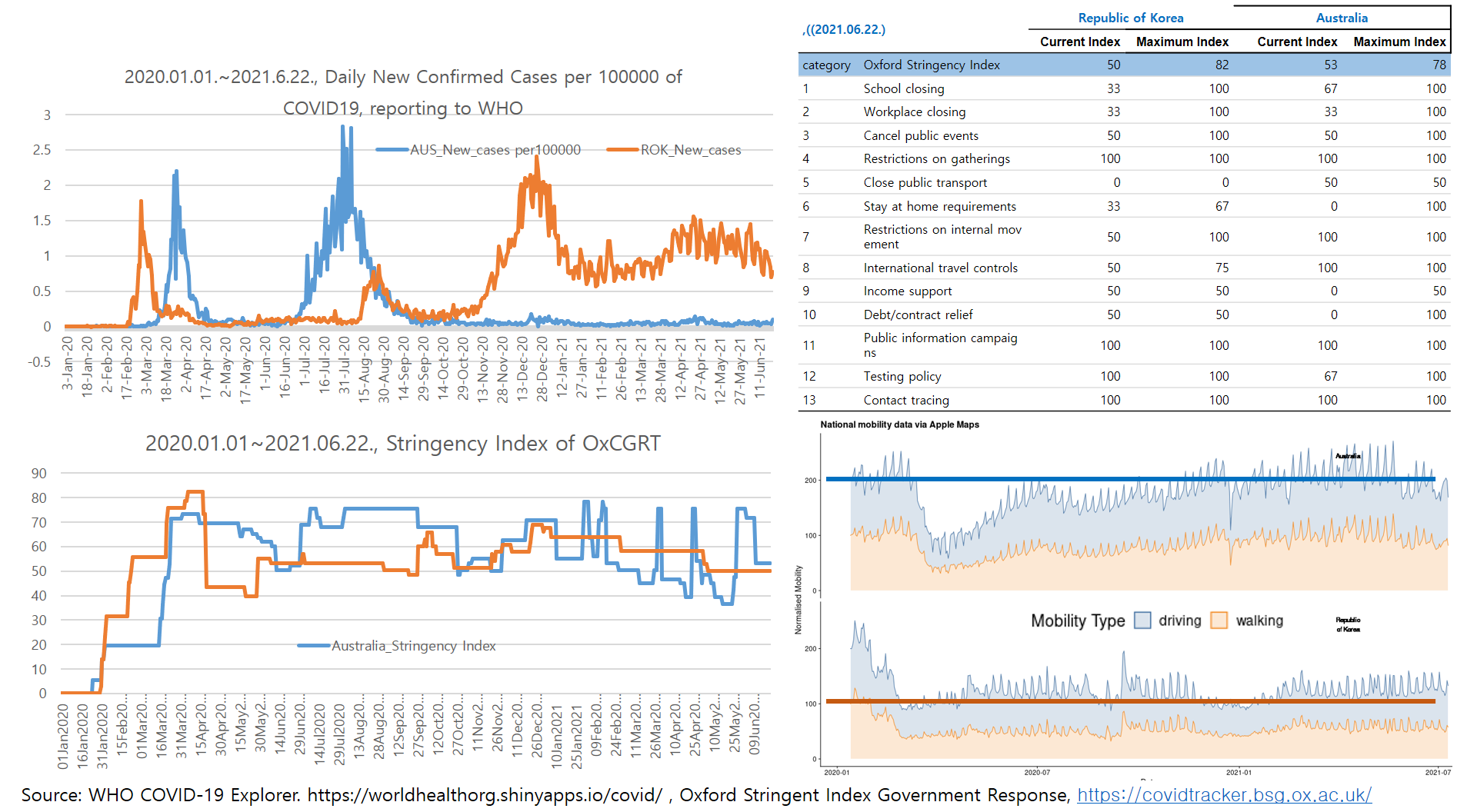
**

Figure 1. Trends of Daily New Confirmed Cases per 100000 of COVID19, Stringency Index of OxCGRT, and Mobitiy via Apple Maps in Australia and Republic of Korea

3. 효과적인 방역정책에 대한 제언

Covid-19 감염은 대유행 이후 1년이 경과하지도 않은 시기에 이미 중증 심·뇌혈관질환의 위험요인(risk Factor)으로 확인되고 있다(11).

SARS-CoV2 변이는 예상보다 너무 빠르고, 백신은 희망과 달리 효과 지속이 짧을 가능성이 있다. 전파력이 변이 출현마다 증가해서 현재 백신으로 퇴치수준의 집단면역을 형성할 수 없고, 백신 효과 제한으로 반복 접종해야 하나 이는 이상반응 증가를 일으킬 수 있는 딜레마에 처해 있다.

COVID19의 질병부담이 급성기에 국한하지 않고, COVID19의 장기 질병부담과 예방접종 후 이상반응의 장기 영향이 불확실한 상태애서 사회가 취할 수 있는 대안은 사회적 거리두기 등의 부작용과 백신 접종 회수를 최소화하면서 감염량을 최소화하고, COVID-19 감염에 의한 직∙간접, 장∙단기 질병부담을 최소화할 수 있는 방안으로 추진되어야 한다. 사회방역은 호주에서 시행한 것과 같이 최고 강도의 조치를 2주 이내로 짧게 시행하고 이 후 2개월 정도 국민들이 비교적 자유롭게 활동할 수 있도록 하는 intermittent mitigation이 보건학적/사회경제적 관점에서 최선의 방안으로 생각된다.

Figure 2는 최근 재증가하는 COVID19 발생과 다가오는 겨울철 방역에 대비하여 필요한 최고강도의 intermittent mitigation 시행에 대한 저자의 주장을 도식화한 것이다.

Red solid line은 부분적인 mittigation을 시행할때의 유행을 예상한 내용이고 blue dot line 은 mittigation을 시행하지 않았을 때의 유행을 예상한 내용이다. Red dot line은 mittigationgn 관리가 잘 되었을 때를 예상한 것이다. Blue solid line 은 intermittant mitigation 을 실시하여 유행을 통제하고 있는 호주의 사례를 표시하였다.

호주의 사례를 참고로 하여 우리나라에서 겨울철에 대비한 방역대책으로 검토할수 있는 방법으로 4단계 방법을 제안한다.

(1) 우선 최고강도의 Mitigation 조치(Everybody, stay home, 가장 강한 Mitigation)를 중재효과가 나타날 수 있는 최소 기간만큼 (COVID-19 SI 4~6일 이상) 전면적으로 실시하여 잔존 감염량을 최대한 줄인다.

(2) 다음단계로 부작용이 큰 사회적 거리두기를 해제하고 사실상 COVID-19 발생 이전과 근접한 수준으로 사회 교류를 복원하고, COVID-19 발생이 일정 기준(예, 일발생 인구 10만명당 1) 될 때까지 지속한다.

(3) 사회 교류 복원 기간이 최소 2~3개월이 될 수 있도록, 조기발견을 통한 Containment 방역 강화와 마스크 상시 착용 등의 예방관리 수단을 동원하여 COVID-19 발생 증가 속도를 최대한 억제한다.

(4) COVID-19 발생이 일정 기준 이상이 되면 다시 최고강도의 Mitigation 조치를 취하고 사회 교류를 복원한다.

백신 접종도 장기간 지속되는 접종 대신 백신을 충분히 확보한 이후 시행기간을 최대한 짧게 시행하는 것이 바람직하다. 한국은 1개월에 5천만 회 이상 접종이 가능한 인프라를 갖추고 있기 때문에 가능한 정책이다. 이로서 집단면역 지속 기간과 효과를 극대화하고, 반복 접종 회수를 최대한 줄일 수 있을 것이다.

**
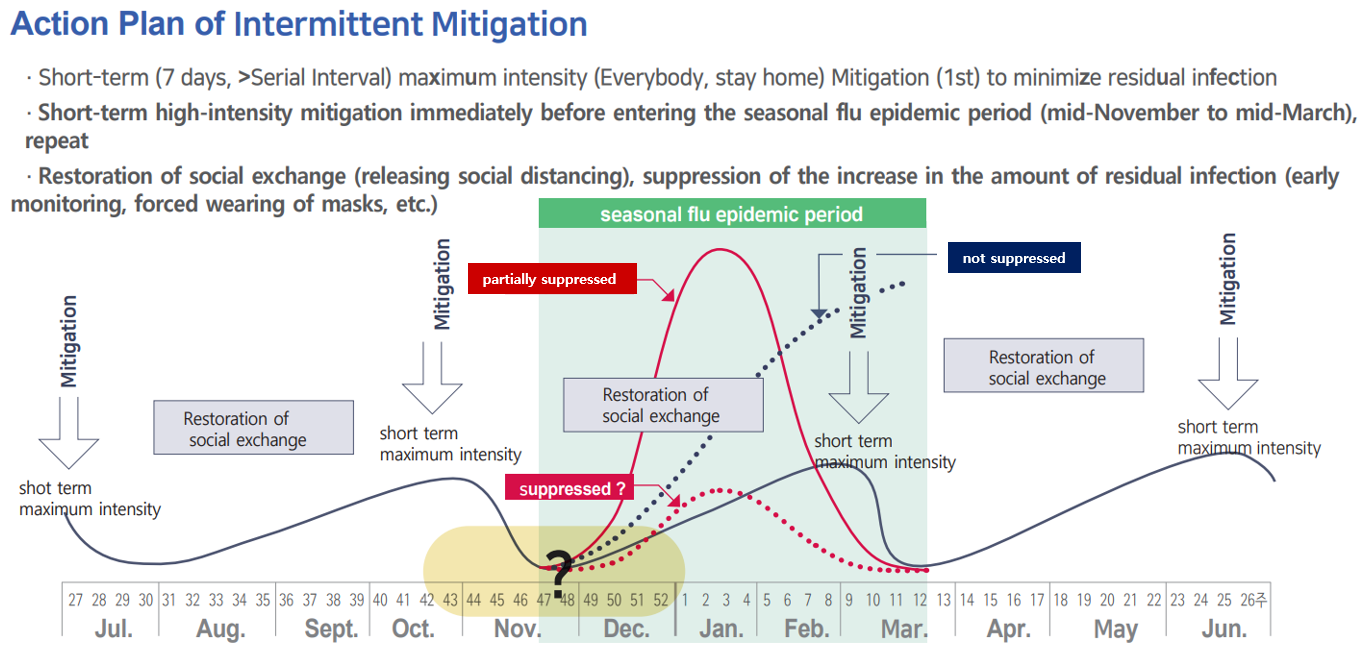
**Figure 2. Action Plan of Intermittent Mitigation.

4. 제한점

호주와 한국은 예방접종률 등은 유사하나 게절과 전체 인구수, 인구밀도, 사회적 행태 등이 다른나라이다. 따라서 두나라를 평면적으로 비교하고 벤치마킹하여 대안을 제시하는 것은 정확성 등에서 많은 제한점이 있다. 이러한 제한점에도 불구하고 호주의 성공사례를 소개 함으로써 한국의 방역정책에 참고로 제시하고자 하였다.

**5. 결론**

한국은 의료기반이 잘 갖추어져 한달에 5천만 접종이 가능하기 때문에 충분한 백신을 확보하여 단기간에 집단접종을 시행할 수 있다. 이 방법으로 단기간에 집단면역을 이룰 수 있으며 재접종을 최소화 할 수 있다. 접종과 함께 높은 강도의 사회적 통제를 단기간 시행하고 사회적 거리두기를 완화하면 겨울철 재유행에 대비하는 효율적 방안이 될 것이다.

**References**

1. Schuppert A, Polotzek K, Schmitt J, Busse R, Karschau J, Karagiannidis C. Different spreading dynamics throughout Germany during the second wave of the COVID-19 pandemic: a time series study based on national surveillance data. Lancet Reg Health Eur 2021 27;6:100151.

2. Davies NG, Abbott S, Barnard RC, Jarvis CI, Kucharski AJ, Munday JD, et al. Etimated transmissibility and impact of SARS-CoV-2 lineage B.1.1.7 in England. Science 2021;372:eabg3055.

3. Allen H, Vusirikala A, Flannagan J, Twohig K, Zaidi A: COG-UK Consortium, et al. Increased household transmission of COVID-19 cases associated with SARS-CoV-2 variant of concern B. 1.617. 2: a national case-control study; 2021 [cited 2021 Jul 1]. Available from: https://khub.net/documents/135939561/405676950/Increa

sed+Household+Transmission+of+COVID-19+Cases+-+national+

case+study.pdf/7f7764fb-ecb0-da31-77b3-b1a8ef7be9aa.

4. Thompson RN, Hill EM, Gog JR. SARS-CoV-2 incidence and vaccine escape. Lancet Infect Dis 2021;21:913-914.

5. Edridge AW, Kaczorowska J, Hoste AC, Bakker M, Klein M, Loens K, et al. Seasonal coronavirus protective immunity is short-lasting. Nat Med 2020;26:1691-1693.

6. Kissler SM, Tedijanto C, Goldstein E, Grad YH, Lipsitch M. Projecting the transmission dynamics of SARS-CoV-2 through the postpandemic period. Science 2020;368:860-868.

7. Mulheirn I, Alvis S, Insall L, Browne J, Palmou C. A sustainable exit strategy: managing uncertainty, minimising harm; 2020 [cited 2021 Jul 1]. Available from: https://institute.global/policy/sustainable-exit-strategy-managing-uncertainty-minimising-harm.

8. Hale T, Angrist N, Kira B, Petherick A, Phillips T, Webster S. Variation in government responses to COVID-19: version 6.0; 2020 [cited 2021 Jul 1]. Available from: https://www.bsg.ox.ac.uk/sites/default/files/2020-05/BSG-WP-2020-032-v6.0.pdf.

9. World Health Organization. WHO coronavirus (COVID-19) dashboard [cited 2021 Jul 26]. Available from: https://covid19.who.int/who-data/vaccination-metadata.csv.

10. Hale T, Webster S, Petherick A, Phillips T, Kira B. Oxford COVID-19 government response tracker [cited 2021 Jul 1]. Available from: https://covidtracker.bsg.ox.ac.uk/.

11. Pero A, Ng S, Cai D. COVID-19: a perspective from clinical neurology and neuroscience. Neuroscientist 2020;26:387-391.
